# Acknowledgments

**Published:** 1859-05

**Authors:** 


TO DR. PREYSS, of Vienna, the able editor of the *Oesterrichische Zeitschrift für Praktische Heilkunde*, we are under obligations for numbers

40–52 of his journal for 1857, and numbers 1–26 for 1858.

To DR. BROWN-SÉQUARD we also tender our thanks for favors extended to our journal in Paris.

